# Hydrogen Gas Inhalation Attenuates Acute Impulse Noise Trauma: A Preclinical In Vivo Study

**DOI:** 10.1177/00034894221118764

**Published:** 2022-08-12

**Authors:** Pernilla Videhult Pierre, Anette Fransson, Marta A. Kisiel, Göran Laurell

**Affiliations:** 1 Division of Audiology, Department of Clinical Science, Intervention and Technology, Karolinska Institutet, Huddinge, Sweden; 2 Department of Surgical Sciences, Uppsala University Hospital, Uppsala, Sweden; 3 Department of Neuroscience, Karolinska Institutet, Stockholm, Sweden; 4 Department of Medical Sciences, Occupational and Environmental Medicine, Uppsala University Hospital, Uppsala, Sweden

**Keywords:** auditory brainstem response, auditory hair cell, guinea pig, hearing loss, noise-induced, molecular hydrogen, otoprotection

## Abstract

**Objective:**

Molecular hydrogen (H_2_) has shown therapeutic potential in several oxidative stress-related conditions in humans, is well-tolerated, and is easily administered via inhalation.The aim of this preclinical in vivo study was to investigate whether impulse noise trauma can be prevented by H_2_ when inhaled immediately after impulse noise exposure.

**Methods:**

Guinea pigs (n = 26) were subjected to impulse noise (n = 400; 156 dB SPL; 0.33/s; n = 11; the Noise group), to impulse noise immediately followed by H_2_ inhalation (2 mol%; 500 ml/min; 1 hour; n = 10; the Noise + H_2_ group), or to H_2_ inhalation (n = 5; the H_2_ group). The acoustically evoked ABR threshold at 3.15, 6.30, 12.5, 20.0, and 30.0 kHz was assessed before and 4 days after impulse noise and/or H_2_ exposure. The cochleae were harvested after the final ABR assessment for quantification of hair cells.

**Results:**

Noise exposure caused ABR threshold elevations at all frequencies (median 35, 35, 30, 35, and 35 dB SPL, the Noise group; 20, 25, 10, 13, and 20 dB SPL, the Noise + H_2_ group; *P* < .05) but significantly less so in the Noise + H_2_ group (*P* < .05). Outer hair cell (OHC) loss was in the apical, mid, and basal regions 8.8%, 53%, and 14% in the Noise group and 3.5%, 22%, and 1.2% in the Noise + H_2_ group. The corresponding inner hair cell (IHC) loss was 0.1%, 14%, and 3.5% in the Noise group and 0%, 2.8%, and 0% in the Noise + H_2_ group. The difference between the groups was significant in the basal region for OHCs (*P* = .003) and apical (*P* = .033) and basal (*P* = .048) regions for IHCs.

**Conclusions:**

Acute acoustic trauma can be reduced by H_2_ when inhaled immediately after impulse noise exposure.

## Introduction

Although occupational noise exposure can be reduced by stricter legislation and personal hearing protection devices,^1^ noise-induced hearing loss (NIHL) still remains a significant occupational health hazard.^2^ Acoustic overexposure can impair the outer hair cells (OHCs), the inner hair cell (IHC) ribbon synapses, the supporting cells in the organ of Corti, the marginal cells of the stria vascularis, and the spiral ganglion cells.^3-6^ The underlying mechanisms include inflammation and oxidative stress caused by a series of complex metabolic alterations in the cochlea.^3,6^ Current treatment of hearing loss is hearing aids and cochlear implants, while established pharmacological treatment to reduce or prevent NIHL in clinical practice is lacking. Human trials on NIHL have demonstrated promising results for several agents, including glucocorticoids, alpha-lipoic acid, carbogen, vitamin B12, and Mg-aspartate, but their clinical value remains unclear.^6,7^ One challenge in drug treatment of the inner ear is the blood-perilymph and the intrastrial fluid-blood barriers^8,9^ that prevent drugs in the systemic circulation from accessing the cochlear compartments. A gaseous agent may circumvent these barriers due to its ability to diffuse through tissues across a concentration gradient.^10^ A gaseous agent may also be inhaled, which enables its immediate and non-invasive delivery at work places without any assistance of medical personel. Molecular hydrogen (H_2_) is gaseous at ambient conditions, is generally considered non-toxic, and has antioxidant and anti-inflammatory properties.^11^ These properties make H_2_ an interesting drug candidate for treatment of acute acoustic trauma in humans. In patients, H_2_ inhalation has shown efficacy in several medical conditions involving oxidative stress and inflammation, including post-acute COVID-19,^12^ chronic obstructive pulmonary disease,^13^ and myocardial^14^ and cerebral^15^ infarctions. Aqueous H_2_, which appears to be more studied, has shown therapeutic potential in the clinic in additional conditions, including gastroesophageal reflux disease,^16^ rheumatoid arthritis,^17^ and in patients with cancer subjected to radiotherapy.^18^ Within audiology, one clinical study and several preclinical studies on H_2_ have been conducted. The clinical study indicated that inhalation of a mixture of H_2_ and oxygen (O_2_) ≥ 3 hours a day for >4 weeks reduced conductive and sensorineural post-radiotherapy hearing loss.^19^ H_2_’s otoprotective potential was initially shown when H_2_-supplemented culture medium increased the survival of hair cells exposed to antimycin A, an effect that was attributed to reduced hydroxyl radical production.^20^ Shortly after, pretreatment with aqueous H_2_ given orally against continuous noise^21^ and given intraperitoneally against impulse noise^22^ were shown to protect the functional hearing of guinea pigs. Using a combination of pretreatment and posttreatment with intraperitoneal aqeous H_2_ against continuous noise, the protective effects of H_2_ was later shown to involve antioxidant^23,24^ and anti-inflammatory effects.^24^ The aim of the present preclinical study was to investigate whether acute acoustic trauma can be prevented by H_2_ when inhaled immediately after impulse noise exposure.

## Materials and methods

### Experimental overview

Guinea pigs were randomly divided into 3 groups: Noise (n = 11), Noise + H_2_ (n = 10), and H_2_ (n = 5). The Noise and the Noise + H_2_ groups were unilaterally exposed to 400 short sound impulses (~156 dB SPL) for approximately 3 min. The Noise + H_2_ and H_2_ groups were subjected to 1-hour H_2_ inhalation, which for the Noise + H_2_ group occurred within minutes after noise exposure. Auditory trauma was assessed by measuring the frequency specific acoustically evoked auditory brainstem responses (ABRs) and hair cell loss 4 days after noise and/or H_2_ exposure.

### Animals

Duncan-Hartley guinea pigs (Lidköpings Kaninfarm, Lidköping, Sweden) of both sexes weighing 260 to 400 g were used. They had normal tympanic membranes and hearing at baseline as determined by otoscopic examination and ABR assessment. They were kept in an enriched environment in small groups with lights on between 7 a.m. and 7 p.m. at a temperature of 21°C and a humidity of 60% with free access to water and standard chow. General anesthesia was achieved with intramuscular ketamine (40 mg/kg b.w.; Ketalar, 50 mg/ml; Pfizer AB, Sweden) and xylazine (10 mg/kg b.w.; Rompun, 20 mg/ml; Bayer Health Care AG, Denmark). Its depth was determined by measurement of the pedal reflex, and additional doses of ketamine (25 mg/kg b.w.) were given if needed. Local anesthesia was achieved with subcutaneous bupivacaine (Marcain, 2.5 mg/ml, AstraZeneca, Sweden). All experimental procedures were performed under anesthesia and aseptic conditions and in accordance with the ethical guidelines of Uppsala University and Swedish regulations for animal care and use (ethical permit C106/13, Uppsala’s ethical committee on animal experiments).

### Noise and H_2_

Noise exposure was performed as previously described.^25^ Briefly, short sound impulses (n = 400; ~156 dB SPL; rate 0.33/s) was generated by the sound card of a notebook computer. The output of the sound card was attached to a noise delivery system with a power amplifier connected to a loudspeaker and a cone-shaped horn. During noise stimulation, the narrow end of the horn was placed at the entrance of the left external ear of the animal using a 5 cm plastic tube. The noise level was calibrated with a Brüel & Kjaer model 4135 condenser microphone and a Brüel & Kjaer 2610 sound level meter before the experiment started.

H_2_ was administered through a facial mask over 1 hour using a gas mixture of H_2_ (2 mol%), oxygen (O_2_; 21 mol%), and nitrogen (N_2_; 77 mol%; AGA Gas AB, Sweden). The flow rate was set at 500 ml/min using a single-stage pressure regulator (C 200/1 A B 3BAR DIN 1, Linde AG, Linde Gases Division, Germany).

### ABR

Acoustically evoked frequency specific ABR recordings at 3.15, 6.30, 12.5, 20.0, and 30.0 kHz were used to measure the functional auditory trauma. Recordings were performed before and 4 days after noise and/or H_2_ exposure. The stimulus signal was generated through a signal analyzer (Tucker-Davis Technologies, FL, USA) controlled by a PC and presented through an electrostatic speaker (EC1; Tucker-Davis Technologies, FL, USA). The speaker was connected to a 10-cm tube positioned in the ear canal of the guinea pig, which was situated in a sound proof box. Neural responses were collected using 3 subdermal electrodes each placed at the vertex (active), the mastoid (reference), and the lower back (ground). The ABR threshold was defined as the lowest stimulus intensity that produced a reproducible response for ABR wave II visualized at the same latency after an average of 1000 recordings. More details are given elsewhere.^25^

### Morphology

OHC loss was used as a measure of morphological auditory trauma. After the final ABR recording, the animal was euthanized with an overdose of sodium pentobarbital (80-100 mg/kg i.p.). The temporal bones were immediately removed and the bullae were opened to expose the cochleae. Small openings were made in the round window and apex, and 4% paraformaldehyde in phosphate buffered saline (pH 7.4; PBS) was gently perfused through the cochlea. Surface preparation was performed as described previously.^26^ Briefly, the bone was gently removed from the organ of Corti, the stria vascularis, the spiral ligament, and the tectorial membrane. The tissue was rinsed in PBS several times, incubated in a solution of 1% bovine serum albumin and 0.3% Triton- X100 for 10 minutes, rinsed, incubated with fluorescent-labeled phalloidin (TRITC 1:200, Sigma-Aldrich) for 45 minutes, and then thoroughly rinsed. The organ of Corti was thereafter dissected in approximately 3-mm-long sections. The sections were placed in glycerol on microscope slides, covered with a coverslip, and sealed with nail polish. All OHCs and IHCs were examined using a Zeiss Axio Observer.Z1 microscope (×40 objective; Carl Zeiss, Germany). A reticule placed in the focus of the microscope eyepiece allowed for 0.25 mm of the coil to be viewed and analyzed at a time. After analyzing all hair cells and scar formations, the percentage of hair cell loss per millimeter and row was calculated. Loss of hair cells was quantified in the left cochlea, that is, the side which was exposed to noise, and in the Noise and Noise + H_2_ groups.

### Data analysis

The frequency specific ABR thresholds in the left ear within each group before noise and/or H_2_ exposure were compared to that 4 days after the exposure using a Wilcoxon’s signed rank test. The frequency specific ABR threshold shifts of the groups were compared using a Mann–Whitney *U* test. The percentage loss of OHCs and IHCs in the Noise and Noise + H_2_ groups was compared by calculating each of the mean OHC loss and the mean IHC loss in the basal, mid, and apical parts of the cochlea, each defined as 1 to 8, 9 to 14, and 15 to 18 mm from the round window. A Mann–Whitney *U* test was used to determine whether the intergroup differences in percent hair cell loss were significant. The statistical analyses were carried out in SPSS for Mac (v 25, 64-bit edition) using an alfa level of 0.05. Exact 2-tailed P values were used. Non-parametric tests were used as the sample sizes were not large enough to compensate for the fact that the data deviated from normal distribution.

## Results

### ABR

**Figure 1.**
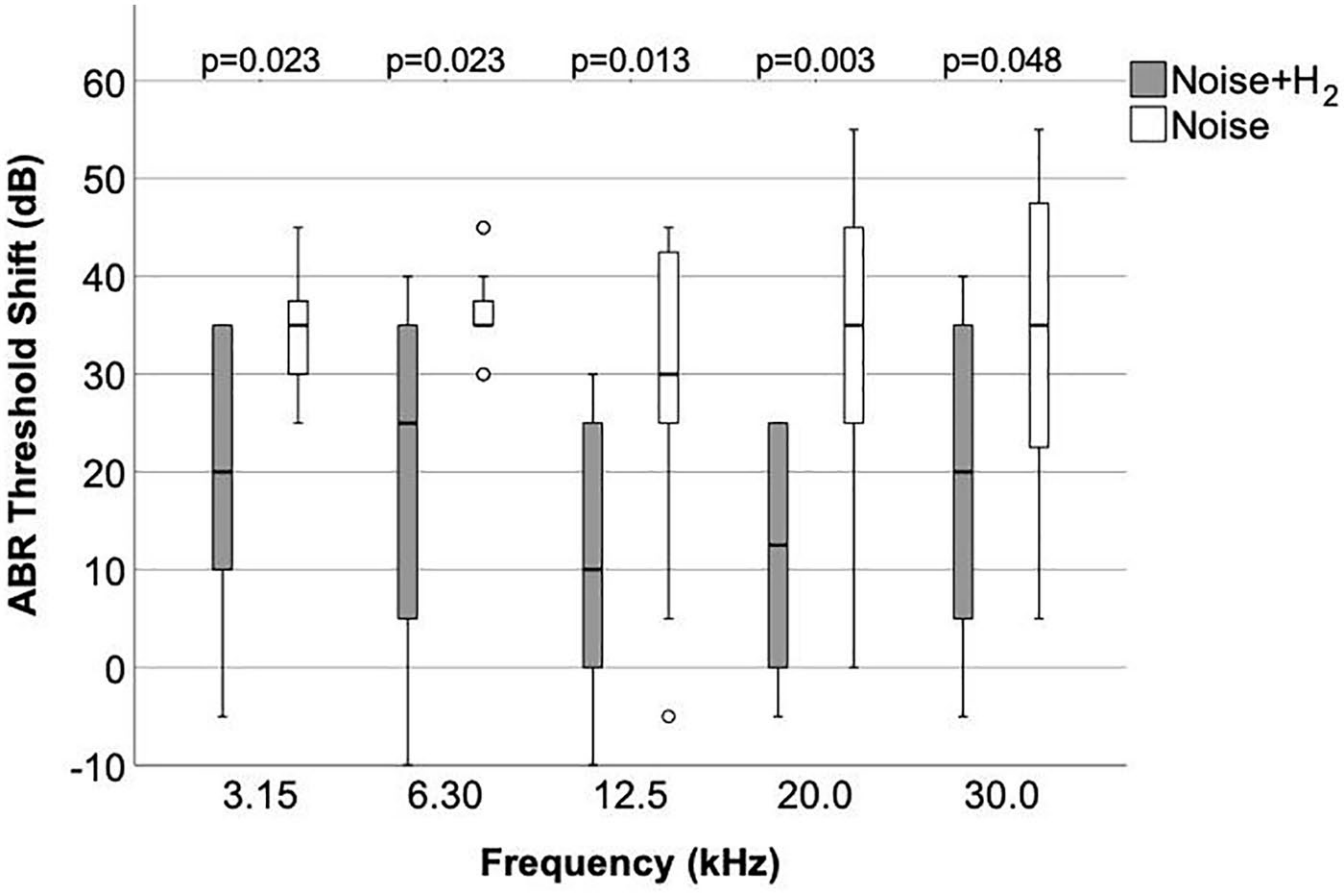
Guinea pigs were exposed to unilateral (left-side) impulse noise (400 short sound impulses; ~156 dB SPL; rate 0.33/s) only (the Noise group; n = 11) or followed by 1-hour H_2_ inhalation (Noise + H_2_; n = 10). Frequency specific acoustically evoked auditory brainstem response (ABR) thresholds of the left ear at 3.15, 6.30, 12.5, 20.0, and 30.0 kHz were determined before and 4 days after the exposure. The frequency specific ABR threshold shifts in each group are shown. The horizontal line in the middle of the boxes represents the median, and the bottom and the top of the boxes represent the first and third quartiles, respectively. The bottom and top whiskers extend to the minimum and maximum values, respectively, that are within 1.5 times the height of the box. The empty circles are outliers, that is, values that are without 1.5 times the height of the box. p is the P value for the inter-group difference in frequency specific ABR threshold shift (2-sided Mann-Whitney *U* test). n = 10 in the Noise + H_2_ group and n = 11 in the Noise group at each frequency.

The ABR thresholds for the Noise, Noise + H_2_, and H_2_ groups were similar at baseline (Table 1). Four days after noise and/or H_2_ exposure, the ABR threshold at each frequency was significantly higher than before treatment in the Noise and Noise + H_2_ groups, while it was unchanged in the H_2_ group (Table 1). The ABR threshold shifts for the Noise and Noise + H_2_ groups are presented in Figure 1. A significant difference was found between these 2 groups at all frequencies, in favor of the Noise + H_2_ group (Figure 1).

**Table 1.** Acoustically Evoked Auditory Brainstem Response Thresholds.

				ABR threshold (dB SPL)				
				Median	Q1	Q3	n	*P* value*
Frequency (kHz)	3.15	Noise	Pre	25	25	35	11	.001
			Post	65	60	65	11	
		Noise + H_2_	Pre	30	25	35	10	.008
			Post	53	35	60	10	
		H_2_	Pre	25	25	30	5	n.s.
			Post	25	25	25	5	
	6.30	Noise	Pre	30	25	35	11	.001
			Post	65	60	70	11	
		Noise + H_2_	Pre	33	30	35	10	.012
			Post	58	35	65	10	
		H_2_	Pre	25	25	30	5	n.s.
			Post	25	25	25	5	
	12.5	Noise	Pre	25	20	35	11	.003
			Post	55	50	65	11	
		Noise + H_2_	Pre	30	30	35	10	.039
			Post	43	35	50	10	
		H_2_	Pre	25	25	30	5	n.s.
			Post	25	25	30	5	
	20.0	Noise	Pre	25	20	35	11	.002
			Post	60	50	70	11	
		Noise + H_2_	Pre	30	30	35	10	.016
			Post	45	35	55	10	
		H_2_	Pre	30	25	30	5	n.s.
			Post	30	30	30	5	
	30.0	Noise	Pre	25	25	35	11	.001
			Post	60	50	75	11	
		Noise + H_2_	Pre	30	25	30	10	.008
			Post	50	35	60	10	
		H_2_	Pre	30	25	30	5	n.s.
			Post	30	25	30	5	

Frequency specific acoustically evoked auditory brainstem response (ABR) thresholds of the left ear of guinea pigs at baseline (pre) and 4 days after (post) unilateral (left-ear) exposure of impulse noise (n = 400 ; ~156 dB SPL; 0.33/s) without (n = 11; the Noise group) or with subsequent inhalation of molecular hydrogen (H_2_; 2 mol%; 500 ml/min; 1 hour; n = 10; the Noise + H_2_ group) or inhalation of H_2_ without prior noise exposure (n = 5; the H_2_ group). *P value for the intra-group difference between pre and post threshold values (2-sided Wilcoxon’s signed-rank test); dB SPL, decibel sound pressure level; n.s., non-significant; Q1, first quartile; Q3, third quartile.

### Hair cell loss

**Figure 2.**
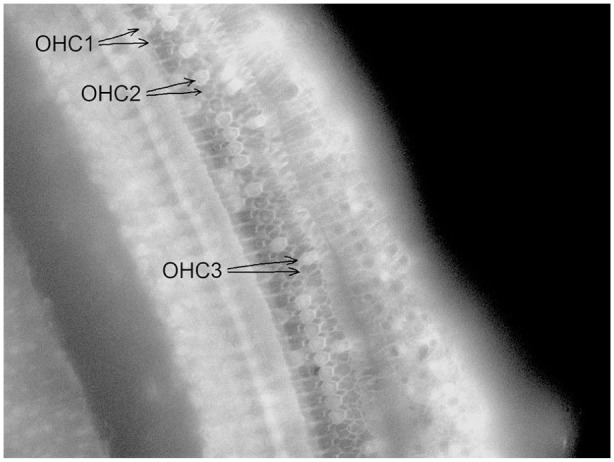
A micrograph of a surface preparation derived from the left cochlea of a guinea pig in the Noise + H_2_ group that shows a remaining (top) and a lost (bottom) outer hair cell (OHC) of the first (OHC1), second (OHC2), and third (OHC3) rows.

**Figure 3.**
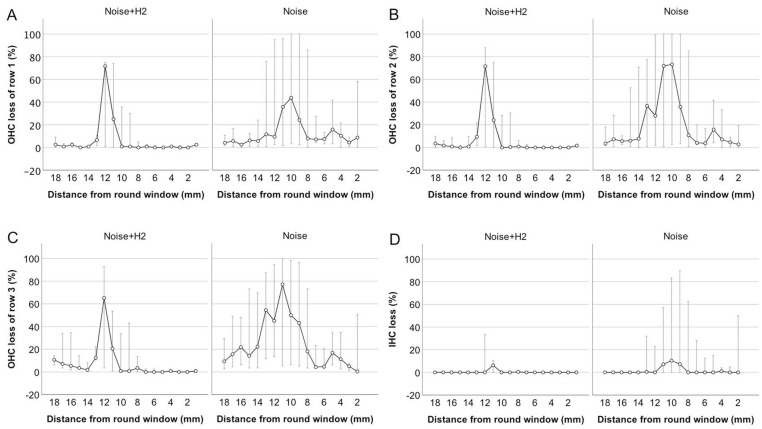
Loss of OHCs in the first (A), second (B), and third (C) rows and loss of inner hair cells (IHCs; (D)) per mm distance from the round window in the left cochlea of the Noise + H_2_ group (n = 10) and the Noise group (n = 11). The circles represent the median, while the bottom and top error bars extend to Q1 and Q3, respectively. The number of observations is 9 at 18 to 10 mm, 8 at 9 and 8 mm, 9 at 7 to 4 mm, 8 at 3 mm, 5 at 2 mm, and 1 at 1 mm in the Noise + H_2_ group and 10 at 18–4 mm, 8 at 3 mm, 4 at 2 mm, 0 at 1 mm in the Noise group.

**Figure 4.**
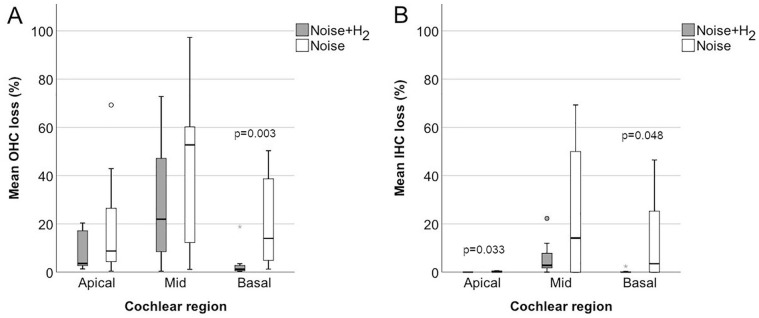
Mean loss of OHCs (A) and IHCs (B) in the apical (15-18 mm), mid (9-14 mm), and basal (1-8 mm) parts of the cochlea in the Noise + H_2_ (n = 10) group and the Noise group (n = 11). The horizontal line in the middle of the boxes represents the median, and the bottom and the top of the boxes represent Q1 and Q3, respectively. The bottom and top whiskers extend to the minimum and maximum values, respectively, that are within 1.5 times the height of the box. The empty circles are outliers, that is, values that are without 1.5 times the height of the box. The grey star is an extreme outlier, that is, a value that is without 3 times the height of the box. *P* is the significant *P* value for the difference in hair cell loss between the treatment groups (2-sided Mann–Whitney *U* test). n = 9 in each region for the Noise + H_2_ group and n = 10 in each region for the Noise group.

Figure 2 gives a micrograph of a surface preparation from an animal in the Noise + H_2_ group, showing the 3 rows of OHCs with some remaining but mostly lost OHCs. The loss of hair cells 4 days after noise exposure per mm distance from the round window is shown in Figure 3A to D. The loss varied greatly between the animals, in particular in the Noise group where the loss also affected a larger area along the cochlea (Figure 3A-D). Taking all 3 OHC rows together, a significant difference between the 2 groups in percentage loss of OHCs was reached in the basal but not the mid or apical parts of the cochlea (Figure 4A). There was also a significant difference between the 2 groups in percentage loss of IHCs in the basal and apical but not the mid parts of the cochlea (Figure 4B). All differences were in favor of the Noise + H_2_ group.

## Discussion

The present study shows that acoustic trauma from impulse noise can be reduced by prompt H_2_ inhalation. To the best of our knowledge, there are 3 previous studies on H_2_ inhalation and noise,^27-29^ and one of them was performed by our group.^28^ In all 3, gaseous H_2_ was administered after continuous noise exposure in guinea pigs. Kurioka et al^27^ found less ABR threshold shifts and OHC loss from 5-hour noise exposure at 121 dB SPL when immediately followed by exposure with gaseous H_2_ 5 hours per day for 5 days. Fransson et al^28^ found less ABR threshold shifts, OHC loss, and damage to IHC synaptic structures from 2-hour noise exposure at 115 dB SPL when immediately followed by the same H_2_ treatment as in the study presented here. Bagheri et al^29^ found less ABR threshold shifts from 6-hour noise exposure at 105 dB SPL per day for 5 days in combination with the asphyxiant carbon monoxide when immediately followed by exposure with gaseous H_2_ (2%) 5 hours per day for 5 days. Similar to those results,^27-29^ acoustic trauma was not fully prevented in the present study. In particular, OHC loss was unsatisfactorily high despite prompt H_2_ treatment. Possibly, a single 1-hour H_2_ inhalation was too short and/or too few. An alternative explanation is that H_2_ inhalation offers limited otoprotection irrespective of treatment schedule. Future studies should establish whether H_2_’s otoprotective effects can be optimized by modifying the duration, timing, and number of H_2_ inhalations. Another relevant parameter for the efficacy is the concentration of H_2_. Kurioka et al^27^ used 3 different H_2_ concentrations and found that 1.0%, and 1.5% but not 0.5% H_2_ were otoprotective. H_2_ is explosive and has a flammability limit of 4% in air. For safety reasons, a H_2_ concentration of 2% H_2_ was used here and in our previous studies on continuous noise^28^ and cisplatin-induced ototoxicity.^30^ H_2_ is however generally considered non-toxic in vivo and has been administered in high concentrations to humans.^11-13,19^ For example, 67% H_2_ was used in the recent audiology study on nasopharyngeal carcinoma patients.^19^

ABR thresholds were assessed at baseline and 4 days after noise exposure and likely include both temporal and permanent threshold shifts.^25,31,32^ ABR threshold shifts were found at all frequencies, in accordance with previous results on the same experimental model for impulse noise exposure.^25^ Hair cell loss was quantified 4 days after noise exposure and may not yet have reached its plateau.^31^ The location of maximum hair cell loss along the cochlea is in accordance with that found in a guinea pig study by Miller and co-workers,^31^ who however used noise-exposure at 120 dB SPL for 5 hours and not impulse noise, but the noise was centered around 4 kHz as in the present investigation. The hair cell loss was more severe and widespread along the cochlea in the Noise group than in the Noise + H_2_ group, which agrees with that described for a more progressive stage of hair cell loss.^31^ To statistically evaluate the hair cell loss, the cochlea was divided into a basal (1-8 mm), a mid (9-14 mm), and an apical (15-18 mm) region as in our previous H_2_ studies.^28,30,33^ A significant difference between the Noise and Noise + H_2_ groups was not reached in the mid region were hair cell loss was most pronounced, likely due to the large variability in hair cell loss in general and in the Noise group in particular with some animals showing high resistance to impulse noise trauma. Overall, the effects of noise varied greatly, both within and between the 2 noise groups. The fact that the susceptibility of noise trauma may vary greatly between individuals has been known for a long time.^34^ In the present study, another possible explanation to the variability in noise trauma also within the groups is that the average noise level reaching the inner ear differed due to inconsistent placement of the sound-transmitting tube. Another possible explanation is sex differences,^35-37^ which was not accounted for as the individual anmal’s sex was unknown. In the Noise + H_2_ group, an additional explanation may be varying dose of inhaled H_2_, as the dose depended on the respiratory rate, which in turn may have differed depending on the depth of anesthesia. In most cases, the ABR results agreed with the hair cell loss also on an individual level. However, in one animal of the Noise + H_2_ group, the hair cell loss was surprisingly low considering the large threshold shifts. Such noise-induced sensorineural hearing loss without hair cell loss may be due to primary neural degeneration.^5^ Loss of hair cells is a well-established but rough method to evaluate the effect of an otoprotective treatment. A higher consistency between auditory function and morphology could perhaps have been achieved if cochlear synaptopathy had been assessed.^5^

The present study did not investigate the underlying mechanisms of H_2_’s otoprotective effects, but they likely involve antioxidative and anti-inflammatory activities as shown previously.^11,23,24,28^ Another possible mechanism is restoration of cochlear blood supply^38,39^ which may be disrupted by noise.^40^

## Conclusion

H_2_ inhalation may reduce auditory trauma from impulse noise in experimental animals. The possibility to optimize H_2_’s otoprotective efficacy and to reduce acute noise trauma in humans by H_2_ inhalation should be investigated in future studies.
